# Batch Studies of Phosphonate and Phosphate Adsorption on Granular Ferric Hydroxide (GFH) with Membrane Concentrate and Its Synthetic Replicas

**DOI:** 10.3390/molecules25215202

**Published:** 2020-11-09

**Authors:** Tobias Reinhardt, Adriana Noelia Veizaga Campero, Ralf Minke, Harald Schönberger, Eduard Rott

**Affiliations:** Institute for Sanitary Engineering, Water Quality and Solid Waste Management (ISWA), University of Stuttgart, Bandtäle 2, 70569 Stuttgart, Germany; an.veizaga@gmail.com (A.N.V.C.); ralf.minke@iswa.uni-stuttgart.de (R.M.); harald.schoenberger@iswa.uni-stuttgart.de (H.S.); eduard.rott@posteo.de (E.R.)

**Keywords:** calcium, (hydrogen-)carbonate, DTPMP, HEDP, NTMP, precipitation

## Abstract

Phosphonates are widely used as antiscalants for softening processes in drinking water treatment. To prevent eutrophication and accumulation in the sediment, it is desirable to remove them from the membrane concentrate before they are discharged into receiving water bodies. This study describes batch experiments with synthetic solutions and real membrane concentrate, both in the presence of and absence of granular ferric hydroxide (GFH), to better understand the influence of ions on phosphonate and phosphate adsorption. To this end, experiments were conducted with six different phosphonates, using different molar Ca:phosphonate ratios. The calcium already contained in the GFH plays an essential role in the elimination process, as it can be re-dissolved, and, therefore, increase the molar Ca:phosphonate ratio. (Hydrogen-)carbonate ions had a competitive effect on the adsorption of phosphonates and phosphate, whereas the influence of sulfate and nitrate ions was negligible. Up to pH 8, the presence of Ca^II^ had a positive effect on adsorption, probably due to the formation of ternary complexes. At pH > 8, increased removal was observed, with either direct precipitation of Ca:phosphonate complexes or the presence of inorganic precipitates of calcium, magnesium, and phosphate serving as adsorbents for the phosphorus compounds. In addition, the presence of (hydrogen-)carbonate ions resulted in precipitation of CaCO_3_ and/or dolomite, which also acted as adsorbents for the phosphorus compounds.

## 1. Introduction

In recent years, global phosphonate consumption has increased from 56,000 t/yr (1998) to 94,000 t/yr (2012) [1,2]. Phosphonates are complexing agents widely used as antiscalants for softening processes in drinking water treatment [3,4,5]. The resulting membrane concentrate is often disposed of into a receiving water body without any further treatment [6,7,8]. A recent study shows the effects of wastewater on the occurrence of various phosphonates in rivers [9]. A significant increase of adsorbed phosphonates in the sediment, which was due to wastewater discharge, was observed. This is of particular interest, since to date, very little is known about the long-term effects of phosphonates in surface waters. Since UV radiation can promote the degradation of phosphonates to readily available orthophosphate, they can contribute to the eutrophication of water bodies [10,11].

By complexing calcium ions [12] and adsorbing on active growth sites of crystals, phosphonates can inhibit CaCO_3_ precipitation [13,14,15]. Due to their threshold effect, they are effective even in substoichiometric concentrations [16]. It is known that phosphonates can adsorb on granular ferric hydroxides (GFH) [4,17,18] as mono-, bi-, or tridentate complexes [19,20]. Calcium ions, whose precipitation is to be prevented by phosphonates, simultaneously have a positive influence on the adsorption of phosphonates on GFH due to the formation of ternary surface complexes [19,21]. The influence of other ions such as magnesium [17] and sulfate [4] on the adsorption on GFH has also been investigated in previous studies.

So far, however, hardly any studies have been published on the influence of a mixture of different ions usually present in membrane concentrates on the adsorption of phosphonates on GFH. Although various phosphonates with different properties are used as antiscalants, only one phosphonate, nitrilotrimethylphosphonic acid (NTMP), has been examined in these studies. Furthermore, little attention has been paid thus far to the possible impacts of potential precipitation.

In a previous study, Reinhardt et al. [18] compared the adsorption of various phosphonates on four different GFH adsorbents. They showed that phosphonates with different properties, such as molecular size and weight, or the number of phosphonate groups, display different behaviors during adsorption. The pH for phosphonate adsorption on GFH should be close to 6. It may be possible to reuse GFH. The authors concluded that further experiments with real wastewater must be conducted in order to investigate the competing or supporting influence of other ions.

**Figure 1:**
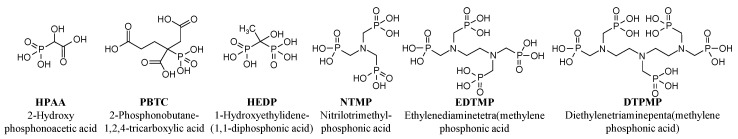
Chemical structures of considered phosphonates.

With the aim of closing the knowledge gaps mentioned above, this study describes batch experiments with the best-performing GFH used in the previous study. In four experiments, the influence of ions present in membrane concentrate on phosphonate adsorption was investigated (mainly focusing on calcium ions). Additional batches that did not include GFH were conducted to investigate whether the observed elimination could be caused by precipitation. For a better understanding of the process, experiments were first performed with synthetic replicas, before conducting subsequent batches with real membrane concentrate. Figure 1 shows the six investigated phosphonates in this study.

## 2. Materials and Methods

### 2.1. Reagents and Chemicals

All solutions were prepared with deionized water made from drinking water in the laboratory, using an ion exchanger (Seradest SD 2000, ELGA LabWater, Celle, Germany) and a downstream filter unit (Seralpur PRO 90 CN).

Acetic acid (AcOH) (100%, Ph. Eur.) and HCl solution (32%, AnalaR NORMAPUR) were obtained from VWR Chemicals (Fontenay-sous-Bois, France). NaNO_3_ (>99%, Ph. Eur.), Na_2_SO_4_ (≥99%, p. a.), CaCl_2_∙2H_2_O (≥99.5%, p. a.), MgCl_2_∙6H_2_O (≥99%, Ph. Eur.), KH_2_PO_4_ (≥99.5%, p. a.), and NaOH (≥99%, Ph. Eur.) were purchased from Merck (Darmstadt, Germany). NaHCO_3_ (≥99%, Ph. Eur.) was purchased from Carl Roth (Karlsruhe, Germany). Ethylenedinitrilotetraacetic acid disodium salt dihydrate (EDTA-Na_2_ dihydrate, Titriplex III, 99–101%, Ph. Eur.) was obtained from Merck (Darmstadt, Germany).

The buffers (2-(*N*-morpholino)ethanesulfonic acid (MES) (≥99%), 3-(*N*-morpholino)propanesulfonic acid (MOPS) (≥99.5%), 4-(2-hydroxyethyl)-piperazine-1-propanesulfonic acid (EPPS) (≥99.5%), 3-*N*-cyclohexylamino-2-hydroxypropanesulfonic acid (CAPSO) (≥99%), and 3-(cyclohexylamino)-1-propanesulfonic acid (CAPS) (≥98%) were purchased from Sigma Aldrich (St. Louis, MO, USA). 

HPAA was purchased from Connect Chemicals (Ratingen, Germany) as a technical solution (50%). PBTC, as a technical solution (50%, CUBLEN P 50), as well as EDTMP (5.3% water of crystallization) and DTPMP (16% water of crystallization), both as solids, were supplied by Zschimmer & Schwarz Mohsdorf (Burgstädt, Germany). HEDP∙H_2_O (≥95%) and NTMP (≥97%) were purchased from Sigma-Aldrich (St. Louis, MO, USA).

### 2.2. Adsorbent

A previous study comparing the adsorption of phosphonates on different granular ferric hydroxides (GFH) showed that FerroSorp RW had the highest adsorption capacity of the compared adsorbents [18]. Therefore, FerroSorp RW from HeGo Biotec GmbH was used in this study. Prior to the experiments, the adsorbent was rinsed once over a sieve with distilled water until the water ran clear to remove GFH dust, and then the GFH was air-dried under a fume hood. The screen mesh width was chosen to ensure a maximum separation of 3 Mass-% of the adsorbent as ultra-fine particles during the rinsing process. The FerroSorp RW that was used had a grain size of 0.5–2.5 mm after rinsing, a point of zero charge (pH_PZC_) of 8.6, and a specific surface area of 210 m^2^/g. According to the manufacturer, it has a calcium content of ≥12–19% (mainly CaCO_3_).

### 2.3. Membrane Concentrate and Its Synthetic Replicas

The membrane concentrate was taken from a low pressure reverse osmosis (LPRO) plant at a public waterworks. It was a clear (0.11 NTU), colorless solution with a low organic load (16.1 mg/L COD). It was characterized by concentrations of 613 mg/L Ca^2+^ and 75 mg/L Mg^2+^ and the associated exceptionally high level of water hardness, combined with a very high buffer capacity (alkalinity = 26.6 mmol/L). The pH value of the sample was approx. 7.9, the electrical conductivity 3.14 mS/cm, the Cl^–^ concentration 229 mg/L, the SO_4_^2–^ concentration 474 mg/L, and the NO_3_^–^ concentration 67.7 mg/L. The total P of the sample was 1.27 mg/L, and the dissolved P fraction (total P fraction of the membrane-filtered sample) was 1.27 mg/L as well. Thus, the particulate P fraction was negligible. The o-PO_4_^3–^-P fraction was 0.53 mg/L, and consequently the organic P fraction was 0.74 mg/L P. The antiscalant (DTPMP) was added to the raw water in the waterworks at a dosage of 0.6 mg/L. With an average nanofiltration yield of 80%, the added antiscalant was concentrated by a factor of five. Thus, the membrane concentrate contains approx. 3 mg/L DTPMP, which corresponds to 0.81 mg/L DTPMP-P. Therefore, it is assumed that the organic P fraction consisted almost exclusively of DTPMP. The experiments were started on the day of sampling.

Synthetic replicas of the membrane concentrate were prepared to investigate the influence of different ions. The concentrations of the compounds in the synthetic replicas matched the concentration of the individual compounds in the membrane concentrate. The composition of the synthetic solutions used is shown under the headings A-N in Table 1, the composition of the membrane concentrate under the heading MC. Preliminary experiments (not described here in detail) showed that the corresponding counterions Na^+^ and Cl^–^ did not have any effect on the adsorption. Since synthetic wastewater lacks the buffering capacity of real wastewater, the addition of an organic buffer (0.01 M) was necessary to keep the pH stable during the adsorption experiments. The influence of the buffers on phosphorus analysis is negligible [22].

### 2.4. Experimental Procedure

Experiments were conducted both with synthetic solutions and with real wastewater. P-containing solutions with different pH values, adjusted with HCl or NaOH, were prepared. For each investigated pH, a buffer was added (target pH value (pH_target_) with buffer concentration in brackets): pH 5 (0.01 M AcOH), pH 6 (0.01 M MES), pH 7 (0.01 M MOPS), pH 8 (0.01 M EPPS), pH 9 (0.01 M CAPSO), pH 10 (0.01 M CAPS), and pH 12 (0.01 M NaOH). 

After the required amount of adsorbent was weighed into a 50 mL centrifuge tube, the tube was filled with the buffered P-containing solution up to the 50 mL mark, immediately capped, and then clamped in the overhead rotator (LLG-uniROTATOR 2) running at 20 rpm. The centrifuge tube was removed after a contact time of seven days, and approx. 20 mL of the supernatant was filtered into an empty glass bottle using a two-part disposable syringe (Norm-Ject, 20 mL, Henke Sass Wolf, Tuttlingen, Germany) with an attachable 0.45 µm nylon filter (Sartorius Stedim Biotech GmbH, Göttingen, Germany). A previous study showed that the relatively long contact time of seven days was necessary to achieve equilibrium [18]. This contact time could have been reduced by grinding the adsorbent or by a higher rotation speed, which would have led to more abrasion of the adsorbent. Since it is known that the particle radius of the adsorbent has an influence on the adsorption behavior [23], the adsorbent should be left as undamaged as possible to allow a better comparison with future column experiments. In a possible future engineering application with filter columns, however, significantly shorter contact times can be achieved, since no equilibrium is necessary in this case. Total P (dissolved), orthophosphate-P, and pH (pH_end_) were determined from the filtrate. As it is possible for pH_end_ to deviate from pH_target_, the figures show pH_end_, which is considered to be more relevant.

Dissolved phosphonates are part of the dissolved unreactive phosphorus (DUP) fraction in wastewater, which corresponds to the difference between dissolved P (total P of membrane-filtered sample) and orthophosphate-P [24]. Therefore, organic P concentrations in mg/L were calculated in accordance with Equation (1).

c(DUP) = c(dissolved P) − c(o-PO_4_-P) (1)

Experiment 1 served to investigate the influence of Ca^II^ on the adsorption of different phosphonates on GFH. Two different molar ratios of Ca:phosphonate, 0:1 and 2:1, at different pH values were considered. Table 2 shows the phosphonate concentrations used in Experiment 1, which was conducted with and without adsorbent to investigate the relevance of precipitation (T = 20 °C; pH 5, 6, 7, 8, 9, 10, and 12; t_c_ = 7 d; initial concentration = 16.1 mg/L HPAA, PBTC, HEDP, NTMP, EDTMP, or DTPMP; Ca:phosphonate 0:1 and 2:1; 0.2 g GFH/L and without GFH).

The objective of Experiment 2 was to examine the influence of Ca^II^ on the adsorption of NTMP and DTPMP at higher Ca^II^ concentrations than in Experiment 1. These two phosphonates were selected as salient representatives of organophosphonates, as they are widely used in industrial applications, and the membrane concentrate investigated in this study contained DTPMP. The experiment was performed at seven different pH values and seven different molar ratios of Ca:phosphonate. These were: 0:1, 1:1, 2:1, 3.67:1, 5:1, 7.33:1, and 18.33:1. The adsorption process was investigated both with FerroSorp RW and without adsorbent to investigate the relevance of precipitation (T = 20 °C; pH 5, 6, 7, 8, 9, 10, and 12; t_c_ = 7 d; initial concentration = 16.1 mg/L NTMP or DTPMP; Ca:phosphonate 0:1–18.33:1; 0.2 g GFH/L and without GFH).

The aim of Experiment 3 was to investigate at which phosphonate and Ca^II^ concentrations and molar Ca:phosphonate ratios phosphonates can be removed without the dosing of GFH, and which factors are decisive in this process. At pH 8 and 9, the NTMP and DTPMP concentrations 3.22 mg/L, 16.1 mg/L, 32.2 mg/L, and 80.5 mg/L (Table 3) with the molar Ca:phosphonate ratios 0:1, 1:1, 2:1, 5:1, 10:1, 25:1, and 60:1 were examined (T = 20 °C; pH 8 and 9; t_c_ = 7 d; initial concentration = 3.22–80.5 mg/L NTMP or DTPMP; Ca:phosphonate 0:1–60:1; without GFH). Additionally, the five highest calcium concentrations were investigated using the same procedure, but in the absence of NTMP and DTPMP, to test possible precipitation of CaCO_3_ and Ca(OH)_2_. Ca^2+^ concentrations before (Ca_start_) and after (Ca_end_) the contact time were determined from the filtrate. This experiment was carried out using a duplicate approach. 

Experiment 4 focused on the membrane concentrate from a drinking water treatment plant, and on its synthetic replicas. The membrane concentrate was reproduced synthetically step by step to investigate the influence of different ions (T = 20 °C; pH 5, 6, 7, 8, 9, 10, and 12; t_c_ = 7 d; for the initial concentration see Chapter 2.3; 0.1 g GFH/L and without GFH).

PHREEQC Interactive 3 with the Minteq.v4 database was used to predict possible precipitates. 

### 2.5. Analytical Methods

For phosphorus analysis, a modification of the ISO 6878 [25] method was used (ISO_mini_). The ISO_mini_ molybdenum blue method has been described in detail by Rott et al. [22]. Prior to analysis, all glass materials that came into contact with the sample were thoroughly rinsed with 10% hydrochloric acid and deionized water. Digestion was performed in a HachLange HT200S thermostat. Absorbance measurements were carried out with a Nanocolor UV/VIS II spectrophotometer from Macherey-Nagel. A WTW SenTix 81 pH electrode in combination with the WTW pH91 instrument was used to determine the pH. In order to determine Ca^2+^, DIN 38406-3 was used [26]. A total sample volume of 50 mL of the solution to be analyzed was transferred into an Erlenmeyer flask with a volumetric pipette. A total of 2 mL of 2 M sodium hydroxide solution and a spatula tip of indicator were added to the flask. The solution was titrated with 0.01 M ethylenediaminetetraacetic acid (EDTA) solution while stirring, until the color changed completely.

## 3. Results and Discussion

### 3.1. Experiment 1—Adsorption Behavior of Six Phosphonates in the Presence of and Absence of Ca^II^

**Figure 2:**
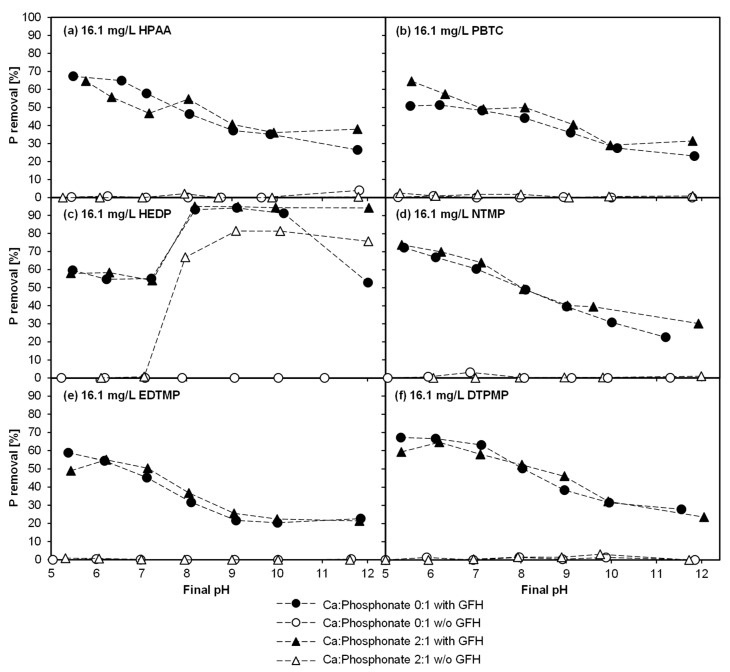
Influence of Ca^II^ presence on phosphonate adsorption (T = 20 °C; t_c_ = 7 d; initial phosphonate concentration = 16.1 mg/L; 0.2 g GFH/L and without GFH). (**a**) 16.1 mg/L HPAA, (**b**) 16.1 mg/L PBTC, (**c**) 16.1 mg/L HEDP, (**d**) 16.1 mg/L NTMP, (**e**) 16.1 mg/L EDTMP, (**f**) 16.1 mg/L DTPMP.

Figure 2 enables a comparison of the adsorption behavior of six different phosphonates at molar Ca:phosphonate ratios of 0:1 and 2:1, with a contact time of seven days and different pH values. Additionally, the removal rates in the absence of GFH are shown, in order to determine the relevance of precipitation. The phosphonates in Figure 2 were sorted in the order of the increasing number of phosphonate groups (PG): HPAA (1 PG), PBTC (1 PG), HEDP (2 PG), NTMP (3 PG), EDTMP (4 PG), DTPMP (5 PG).

For all phosphonates, except HEDP, the removal rates decreased with increasing pH to a similar extent (removal rates at pH_target_ 5 and 12): HPAA from 67% to 27%, PBTC 65% to 23%, NTMP 74% to 23%, EDTMP 59% to 24%, DTPMP 66% to 24%. The difference in the removal rate between the solutions in the presence of and absence of Ca^II^ was always less than 13%. Therefore, no major influence of Ca^II^ on phosphonate removal was observed. This stands in contradiction to several previous studies. Nowack and Stone [21] investigated the adsorption of HEDP, NTMP, EDTMP, and DTPMP on goethite and found that excess Ca^II^ concentrations significantly increased the maximum loading. Even at an equimolar Ca:NTMP ratio, the maximum loading for NTMP almost doubled. Similarly, Boels et al. [4] observed a near doubling of the maximum loading of GFH at a molar Ca:NTMP ratio of 2:1. At a molar ratio of 60:1, it was possible to increase the loading even further. Rott et al. [27] also found a positive influence of Ca^II^ on the adsorption of NTMP and DTPMP on magnetic adsorbent particles (ZnFeZr-oxyhydroxide). In addition, Chen et al. [17] found a positive influence of the hardness ions calcium and magnesium on NTMP adsorption on GFH. The aforementioned studies attribute this behavior mainly to the potential formation of ternary complexes. Ternary complexes can build a bridge between the GFH surface and phosphonates, leading to an increased adsorption [4,19,20].

In the batches without GFH, no removal could be detected. Thus, for this experiment, precipitation can be excluded as the cause of phosphorus removal. HEDP, however, deviated clearly from the behavior of the other phosphonates. In the presence of GFH, it showed a removal of 60% (pH_target_ 5) to 53% (pH_target_ 12), whereas the removal at pH_target_ 8 to 10 was > 90%.

A closer look at the behavior of HEDP reveals two particularly noteworthy aspects. First, with a molar Ca:phosphonate ratio of 2:1, phosphorus was removed even in the absence of GFH, and second, in the presence of GFH, phosphorus was removed without the addition of Ca^II^. In the latter case, about 67% of the phosphorus was removed at pH 8.0, about 81% at pH 9.1 and 10.1, and about 76% at pH 12.0. The removal of phosphorus even without the dosing of GFH suggests precipitation of CaCO_3_ or Ca-HEDP complexes. According to calculations performed with PHREEQC, at a calcium concentration of 156.2 µmol/L, more than 7.2 mmol/L of (hydrogen-)carbonate would be necessary to precipitate CaCO_3_ at pH 8.0. These calculations do not consider HEDP, which complexes at least parts of the Ca^II^ and additionally inhibits precipitation of CaCO_3_ [12,28]. Since deionized water was used in the experiments, and the samples were rotated in closed centrifuge tubes (closed system), the (hydrogen-)carbonate content (from airborne CO_2_) of the solutions is expected to be low. Therefore, the formation of CaCO_3_ is unlikely. 

Another explanation for the observed HEDP elimination in the absence of GFH could be the precipitation of Ca-HEDP complexes. Several researchers have investigated various different calcium-phosphonate precipitates, such as Ca-HEDP [29], Ca-NTMP [16,30], and Ca-DTPMP [31]. However, these studies were conducted using relatively high phosphonate concentrations. In another study, Zhang et al. [32] found more Ca-phosphonate precipitation for HEDP as compared with other phosphonates. The authors concluded that the order of solubility of the Ca-phosphonate complexes was as follows: PBTC > DTPMP > EDTMP > NTMP > HEDP. This corresponds with the results of the current study and the findings of Amjad et al. [33], who found a calcium ion tolerance of PBTC >> HEDP.

Interestingly, between pH 8 and 10, a peak in phosphorus removal was observed without the addition of Ca^II^. The reason for this could be the dissolution of calcium from the GFH. The re-dissolved Ca^II^ could then lead to precipitation. To investigate this in more detail, a 0.01 M CAPSO solution at pH 9 with 0.2 g/L GFH was rotated with a contact time of seven days (without a phosphonate). A Ca^2+^ concentration of 11.0 ± 1.1 mg/L could then be observed in the membrane-filtered supernatant. According to the manufacturer’s statement specifying ≥12–19% calcium content in the GFH, a dosage of 0.2 g/L GFH would result in a maximum calcium concentration in the solution of 24 to 38 mg/L, if completely re-dissolved. Therefore, it can be assumed that approx. 29% to 46% of the Ca^II^ was re-dissolved.

Those measurements may explain why no positive effect of Ca^II^ on the removal of phosphorus was observed with the other five phosphonates. A calcium concentration of 11.0 mg/L (as re-dissolved from the GFH) already corresponds to the following molar Ca:phosphonate ratios: Ca:HPAA 2.7:1, Ca:PBTC 4.6:1, Ca:HEDP 3.5:1, Ca:NTMP 5.1:1, Ca:EDTMP 7.5:1, and Ca:DTPMP 9.9:1. Therefore, although no Ca^II^ was added, the positive effect of Ca^II^ had probably already been achieved by re-dissolution from the GFH. This shows the important role of the CaCO_3_ content of the GFH and also explains the deviation between these results and those found in previous publications.

In conclusion, Experiment 1 yielded two findings: First, the re-dissolved calcium from the GFH had a positive effect on phosphonate adsorption, possibly attributable to the formation of ternary complexes. Second, at the given conditions, HEDP was found to precipitate presumably as Ca-phosphonate complexes, which also increases its elimination rate.

### 3.2. Experiment 2—Adsorption Behavior of NTMP and DTPMP in the Presence of Ca^II^ in Higher Concentrations

**Figure 3:**
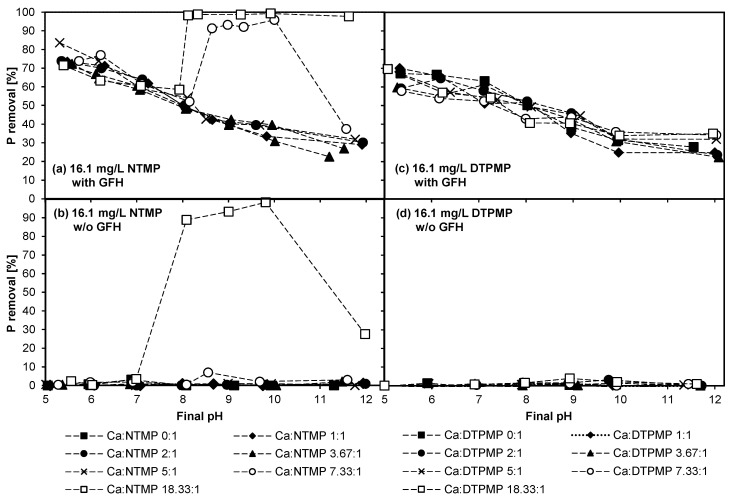
Influence of various molar Ca:phosphonate ratios on phosphonate adsorption (T = 20 °C; t_c_ = 7 d; initial concentration = 16.1 mg/L phosphonate; 0.2 g GFH/L and without GFH). (**a**) 16.1 mg/L NTMP with GFH, (**b**) 16.1 mg/L NTMP without GFH, (**c**) 16.1 mg/L DTPMP with GFH, (**d**) 16.1 mg/L DTPMP without GFH.

The aim of Experiment 2 was to investigate the influence of Ca^II^ in higher concentrations than those in Experiment 1 on the adsorption of NTMP and DTPMP on GFH. Figure 3 shows the results of batches with NTMP and DTPMP in the presence of and absence of GFH over different pH values from 5 to 12.

According to Figure 3b, in the batches without adsorbent, no removal of NTMP took place up to a molar Ca:NTMP ratio of 7.33:1. A different behavior was observed when Ca^II^ was added in a molar Ca:NTMP ratio of 18.33:1. NTMP was removed at particular pH values: ~89% at pH 8.1, ~93% at pH 9.0, ~98% at pH 9.8, and ~28% at pH 12.0. A possible explanation for this behavior could be CaCO_3_ or Ca-NTMP precipitation. According to calculations performed with PHREEQC, more than 0.8 mmol/L of (hydrogen-)carbonate would be necessary at a calcium concentration of 986.3 µmol/L to precipitate CaCO_3_ at pH 8.1. These calculations do not consider NTMP, which complexes at least parts of the Ca^II^ and inhibits precipitation of CaCO_3_ [12,13]. The (hydrogen-)carbonate content of the solutions should be low due to the use of deionized water in the experiments, and due to the rotation of the samples in closed centrifuge tubes (closed system). Thus, the formation of CaCO_3_ is unlikely. 

In the case of added GFH adsorbent (Figure 3a), the adsorption of NTMP was nearly identical at molar Ca:NTMP ratios of up to 5:1. At a molar ratio of 7.33:1, between pH 8.5 and 10.0, and at 18.33:1, between pH 8.1 and 11.6, increased removal occurred. Interestingly, when comparing the batches with (Figure 3a) and without (Figure 3b) GFH, a discrepancy can be observed. If precipitates were the reason for an increased removal, a removal of phosphorus should have also been seen at a molar Ca:NTMP ratio of 7.33:1 from pH 8.5 to 10.0 and at a molar ratio of 18.33:1 at pH 12, as observed in the batches without GFH. However, this can be explained again by the re-dissolution of calcium from the GFH, which may have led to a higher availability of Ca^II^ in the batches with GFH than indicated by the molar ratios in Figure 3.

In Figure 3c,d the behavior of DTPMP is shown. In the batches without GFH (Figure 3d), no removal of DTPMP took place throughout all pH values and all molar Ca:DTPMP ratios tested. Therefore, a precipitation of CaCO_3_ or Ca-DTPMP can be excluded. It is known that DTPMP, unlike other organophosphonates such as HEDP, NTMP, and EDTMP, does not precipitate as 1:1 complexes [34]. The batches with DTPMP and GFH (Figure 3c) show the typical behavior of phosphonates on iron-containing surfaces, namely a decreasing adsorption capacity with increasing pH [3,18,21,35]. The differences in phosphorus removal among the different molar Ca:DTPMP ratios do not show any pattern; therefore, they must have been due to inaccuracies that commonly arise when conducting experiments. Certain deviations in the results of these experiments are to be expected, since some of the input variables may already differ slightly, such as the weighed adsorbent mass, homogeneity of the GFH, pH_end_ value, and so on. Repetitions of selected batches produced similar results (not shown in the figures for better readability). However, despite these deviations, tendencies can still be clearly identified.

In conclusion, the batches with NTMP showed precipitation, which presumably consisted of Ca-NTMP complexes, whereas the batches with DTPMP did not show any precipitation. This corresponds with data found in the literature: Zhang et al. [32] observed that Ca-NTMP complexes have a lower solubility than Ca-DTPMP complexes. Furthermore, Gledhill and Feijtel [36] stated that Ca-NTMP complexes have higher stability constants than Ca-DTPMP complexes at any given pH. Taken together, these conclusions indicate that there are more Ca-NTMP than Ca-DTPMP complexes at any given pH, but the solubility of the Ca-NTMP complexes is lower.

### 3.3. Experiment 3—Investigations on NTMP and DTPMP Precipitation

Since the previous experiments had shown that precipitation is responsible for increased elimination, the aim of Experiment 3 was to investigate this phenomena in more detail. The experiment was necessary because it does not seem to be possible to predict the precipitation of Ca-phosphonate complexes on the basis of the existing data. Depending on the experimental conditions (molar Ca:phosphonate ratio, pH, ionic strength, temperature) different complexes can precipitate [30,31], but reliable solubility products have been published only for some of them. In addition, the presence of calcium can have an effect on the pK_a_ values of the phosphonates [29]. Additionally, a critical evaluation showed that the stability constants of DTPMP are not reliable, due to difficulties in synthesis and purification [34].

Different phosphonate concentrations and molar Ca:phosphonate ratios were applied (Table 4 and Table 5). The pH values 8 and 9 were examined, as the previously described increased removal of phosphonates started in this pH range. Higher pH values were not investigated, because lower pH values are recommended for the adsorption of phosphonates on GFH [18]. In Table 4 and Table 5, removal rates higher than 90% are highlighted in dark gray, and removal rates ≥5% and ≤90% are highlighted in light gray. Removal rates of less than 5% were considered to be measurement inaccuracies, as were negative values (which were within the 5% inaccuracy range). Standard deviations are shown in Tables S1 and S2. Calcium concentrations that are discussed separately are highlighted in bold type.

Table 4 shows the results of the batches with NTMP at different calcium concentrations. In the batches without Ca^II^ and with a molar Ca:NTMP ratio of 1:1, no removal of phosphorus was observed. The same applies to the batches with 3.22 mg/L NTMP at all molar Ca:NTMP ratios. At higher NTMP concentrations, phosphorus was removed almost completely above particular calcium concentrations. At pH 9, the removal started at lower molar Ca:NTMP ratios than at pH 8 (e.g., with 80.5 mg/L NTMP at pH 9, a molar Ca:NTMP ratio of 2:1 was sufficient for an elimination of 91%, whereas at pH 8 no removal occurred).

The three batches with the same Ca^II^ concentration of 0.54 mmol/L highlight an interesting aspect (highlighted in bold type). The NTMP removal at pH 9 increased with increasing NTMP concentration from 64 ± 0.4% at 16.1 mg/L, and 78 ± 1.9% at 32.2 mg/L, up to 91 ± 0.2% at 80.5 mg/L. This indicates a precipitation of Ca-NTMP complexes, since a possible precipitation of CaCO_3_ would have been inhibited by increasing concentrations of NTMP, due to its scale inhibition effect [13].

In Table 5 the results of the DTPMP batches at different calcium concentrations are shown. At molar Ca:DTPMP ratios of up to 2:1 and at 3.22 mg/L DTPMP, no removal of phosphorus was observed. At higher DTPMP concentrations, however, phosphorus was at least partially removed at particular Ca^II^ concentrations. Similar to NTMP, at pH 9, DTPMP was eliminated at lower molar Ca:DTPMP ratios than at pH 8.

At pH 8 and 16.1 mg/L DTPMP, a removal of 54% was observed at a molar Ca:DTPMP ratio of 60:1. At a concentration of 12 mM Ca and 0.73 µM DTPMP (0.42 mg/L) at 70 °C, Kan et al. [31] found a crystalline Ca-DTPMP precipitate. Yan et al. [37] also assumed precipitation of a Ca-DTPMP complex in their experiments with residual concentrations of 0.06 mM DTPMP and approx. 0.8 mM calcium.

An increased removal rate of DTPMP was observed at a constant Ca^II^ concentration of 0.70 mmol/L, paralleled by increasing DTPMP concentrations (Table 5) (i.e., at pH 9; no elimination was found at 16.1 mg/L DTPMP, but at 80.5 mg/L DTPMP, 62 ± 1.9% was eliminated). Moreover, at pH 8, at a Ca^II^ concentration of 1.40 mmol/L and 32.2 mg/L DTPMP, 56 ± 1.4% was removed, whereas at 80.5 mg/L DTPMP, a higher removal rate of 74 ± 0.3% was observed. This indicates that precipitation of Ca-DTPMP complexes occurred, as it was observed for NTMP (Table 4).

The results of Experiment 3 match the results from Experiment 2, in which the same concentration of 16.1 mg/L NTMP and DTPMP was used. In conclusion, NTMP and DTPMP demonstrated similar behavior. However, at comparable molar Ca:phosphonate ratios, NTMP showed a higher removal than DTPMP. This corresponds with the findings of Zhang et al. [32], who found the solubility of Ca-DTPMP complexes to be higher than that of Ca-NTMP complexes.

To gain more knowledge as to which substances are precipitated, further batches were investigated with the five highest Ca^II^ concentrations used in Experiment 3 in the absence of phosphonate. In this case, the calcium concentration was measured in the membrane filtrate after seven days of rotation (Table 6). Ca_start_ deviated only ±3.4% from the target calcium concentration (Ca_target_). Therefore, this deviation is assumed to represent the degree of measurement inaccuracy inherent in this calcium determination method.

The initial and final pH values are nearly identical. A precipitation of CaCO_3_, however, would have resulted in a pH decrease (calculations performed with PHREEQC) that should have been noticeable, even though a buffer was used. The deviation between the initial and final concentration of Ca^2+^ varied between −3% and +3%. The negative deviations are attributable to batches in which the measured final concentration was above the measured initial concentration. As this is not possible, the negative values indicate the measurement error of the analysis method. As the positive deviations are of the same order of magnitude, it is assumed that the deviations within this range are due to measurement inaccuracies, which is also in line with the deviations between Ca_start_ and Ca_target_ as mentioned above. It can be concluded that neither CaCO_3_ nor Ca(OH)_2_ precipitated. Since precipitation only occurred in the presence of the phosphonates, this is another indication that the precipitates were Ca-phosphonate complexes.

### 3.4. Experiment 4—Adsorption Behavior of Membrane Concentrate and Its Synthetic Replicas

#### 3.4.1. Adsorption of DTPMP and Orthophosphate

**Figure 4:**
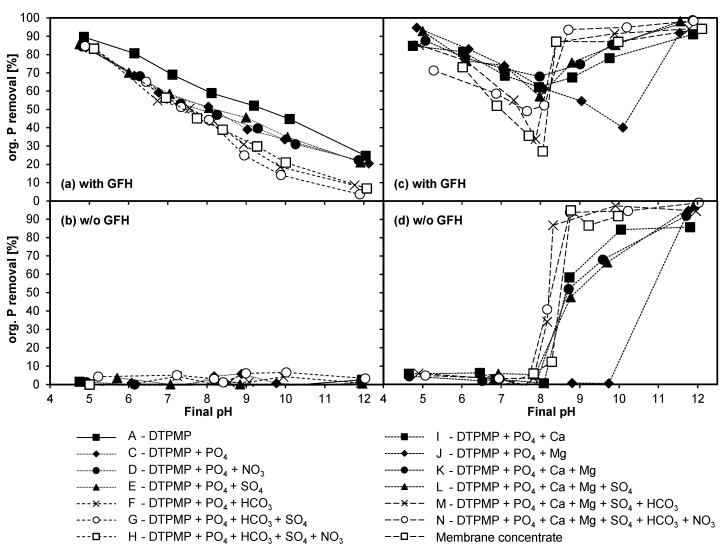
Organic P removal from synthetic replicas and real membrane concentrate (T = 20 °C; t_c_ = 7 d; initial concentrations: see Chapter 2.3; 0.1 g GFH/L and without GFH). (**a**) solutions A, C-H with GFH, (**b**) solutions A, C-H without GFH, (**c**) solutions I-N and membrane concentrate with GFH, (**d**) solutions I-N and membrane concentrate without GFH.

**Figure 5:**
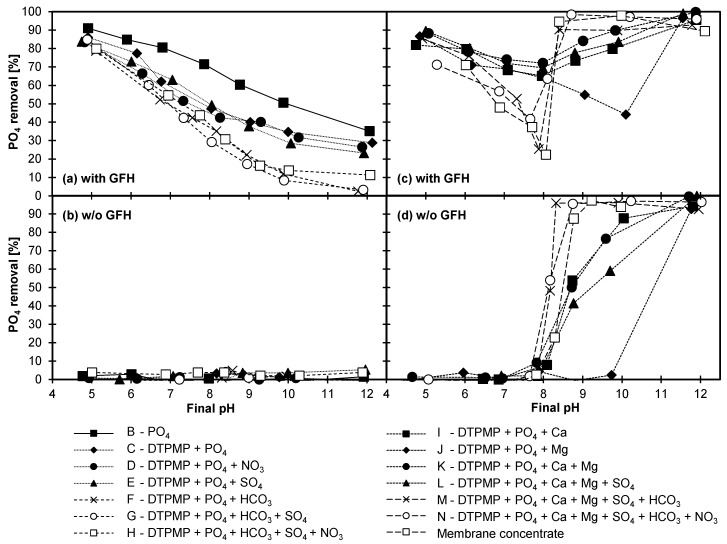
Phosphate removal from synthetic replicas and real membrane concentrate (T = 20 °C; t_c_ = 7 d; initial concentration see chapter 2.3; 0.1 g GFH/L and without GFH). (**a**) solutions B-H with GFH, (**b**) solutions B-H without GFH, (**c**) solutions I-N and membrane concentrate with GFH, (**d**) solutions I-N and membrane concentrate without GFH.

The aim of Experiment 4 was to analyze the respective competitive or synergistic effects of ions on phosphorus removal. To this end, batch adsorption experiments with the real membrane concentrate and its synthetic replicas were conducted. For a better understanding of the results, they have been broken down as follows: Figure 4a,b show the influence of anions in the presence of and absence of GFH, and Figure 4c,d show the influence of cations with and without GFH present. Figure 5 shows the results for orthophosphate with the same layout.

The adsorption of DTPMP on GFH in the presence of negatively charged compounds is depicted in Figure 4a. As observed in previous studies [3,18,21,35], the adsorption capacity decreased with increasing pH. Furthermore, in the mere presence of DTPMP (solution A), the elimination was higher than in all other combinations with anions. By comparing solution A (only DTPMP in the solution) with solution B (only orthophosphate in the solution, Figure 5a), a slightly higher adsorption of orthophosphate becomes apparent. This could be attributed to the large difference in molecular size.

In the presence of DTPMP and orthophosphate (solution C), the elimination of DTPMP was slightly suppressed. This is in line with the findings of Nowack and Stone [35], who observed the suppression of phosphate adsorption in the presence of phosphonates, and the lowering of phosphonate adsorption in the presence of phosphate.

#### 3.4.2. Influence of Anions

When nitrate was added (solution D), no influence was observed. Although nitrate can adsorb on GFH [38], there seems to be no competition for the same adsorption sites. Similarly, Zelmanov and Semiat [39] could not detect any influence of nitrate on the adsorption of orthophosphate on iron oxide/hydroxide nanoparticle-based agglomerates.

The presence of sulfate (solution E, Figure 4a and Figure 5a) also had no observable effect on the adsorption of phosphorus compounds. This is consistent with the results of Boels et al. [40], who found only a minor influence of sulfate on the adsorption of NTMP on waste filtration sand, and with Boels et al. [4], who observed no influence of sulfate ions on NTMP adsorption on GFH. Although only very few investigations have been published on the effect of sulfate on phosphonate adsorption, several studies have investigated the possible competitive adsorption of sulfate and orthophosphate. According to a study by Geelhoed et al. [41], in which the competitive adsorption of sulfate and orthophosphate on goethite was analyzed, phosphate proved to be much more competitive than sulfate, despite the ability of sulfate to form inner-sphere complexes. The presence of sulfate resulted only in a small decrease in phosphorus adsorption at pH < 4. Moreover, Violante et al. [42] also found that sulfate competed only poorly with orthophosphate for adsorption sites of minerals and soils at pH values above 5. Genz et al. [43] could not detect any influence of sulfate ions when a membrane bioreactor filtrate was spiked with sulfate prior to phosphorus removal with GFH. Thus, the results of the current study regarding the influence of nitrate und sulfate ions are in line with those found in the literature.

With the addition of (hydrogen-)carbonate (solution F, Figure 4a and Figure 5a), the adsorption of organic phosphorus decreased slightly at pH 5 to 7, and distinctly from pH 8 to 12. Boels et al. [40] found that (hydrogen-)carbonate ions interfered with the adsorption of NTMP at low concentrations. Another study conducted by Su and Suarez [44] found that (hydrogen-)carbonate lowered the electrophoretic mobility and reduced the pH_PZC_ of iron oxide adsorbents, suggesting inner-sphere carbonate adsorption. Lowering the pH_PZC_ would lead to greater electrostatic repulsion between DTPMP, which is highly negatively charged at high pH values, and GFH. A study investigating the adsorption of orthophosphate on iron oxide/hydroxide nanoparticle-based agglomerates showed a significant impact of (hydrogen-)carbonate concentration. Experiments with an initial P concentration of 10 ppm resulted in a residual P concentration of < 0.05 ppm with no (hydrogen‑)carbonate ions, and of 0.55 ppm in the presence of 1250 ppm (hydrogen-)carbonate ions (pH 7.5) [39]. This is also in line with the findings of Chitrakar et al. [45], who stated the order of selectivity for phosphate adsorption on goethite at pH 8 as Cl^–^, NO_3_^–^, SO_4_^2–^ << CO_3_^2–^, HPO_4_^2–^. A study investigating the adsorption of carbonate found that phosphate has a higher affinity towards ferrihydrite than carbonate. A high concentration of carbonate ions was needed before a significant suppression of phosphate adsorption was observable, which increased with increasing pH [46]. In the current study, such a high concentration of (hydrogen-)carbonate ions was present. The increased competition for adsorption sites at pH > 8 could be attributed to the presence of divalent CO_3_^2–^ ions and the resulting greater electrostatic attraction. The additional presence of sulfate (solution G) or sulfate and nitrate (solution H) resulted in no further reduction of adsorption. Figure 4b and Figure 5b show that, without GFH, there was no noteworthy elimination of organic phosphorus and phosphate. Thus, precipitation can be excluded.

#### 3.4.3. Influence of Cations

Figure 4c,d and Figure 5c,d show the effect of the two hardness ions calcium and magnesium on the removal of DTPMP and phosphate in the presence of and absence of GFH. The figures also illustrate the effect of the two cations in combination with the previously investigated anions up to a near-complete synthetic replication of the membrane concentrate, as well as with the original membrane concentrate.

As shown in Figure 4c and Figure 5c, a noticeable change was observed with the addition of cations, indicating a possible synergistic interaction. The distinct tendency towards a drastically enhanced adsorption at pH values > 8 even resulted in an elimination increase of up to > 60 percentage points (e.g., at pH 12, from ~30% (solution C) to > 90% in the presence of Ca^II^ (solutions I to N)). However, at pH values > 8, a removal was also observable without GFH (Figure 4d and Figure 5d). Thus, precipitation must have occurred in some form at pH > 8.

Interestingly, up to pH 8, the combination of DTPMP, PO_4_-P, and Ca^II^ (solution I) resulted in a higher elimination of organic phosphorus than the combination of only DTPMP and PO_4_-P (solution C). The higher elimination was comparable to the elimination achieved for solution A (mere presence of DTPMP). The same kind of effect was observed with the solution with additional Mg^II^ (solution J, up to pH 10) and the solution with additional Ca^II^ and Mg^II^ combined (solution K). Up to pH 8, however, no removal by precipitation was observed in the absence of GFH (Figure 4d and Figure 5d). Thus, at pH < 8, the enhanced elimination by Ca^II^ and Mg^II^ ions must have been due mainly to the formation of ternary complexes [19]. These results stand in agreement with those of previous studies that have shown that excess Ca^II^ concentrations can substantially increase the maximum surface coverage of phosphonates [4,17,21,27]. Various studies have attributed this behavior to the formation of ternary complexes. Excess magnesium is known to have similar effects [47].

#### 3.4.4. Combined Influence of Anions and Cations

The additional presence of sulfate ions (solution L) did not lead to altered behavior. When (hydrogen-)carbonate was added to solutions with Ca^II^ and Mg^II^ (solutions M and N), up to pH 8, a lower removal of organic phosphorus and PO_4_-P was observed as compared with other solutions containing cations (solutions I to L). This supports the already noted assumption that (hydrogen‑)carbonate is a competing ion for phosphonates.

Above pH 8, on the other hand, even in the absence of GFH, organic phosphorus and PO_4_-P was eliminated (Figure 4d and Figure 5d). This proves that the elimination observed in this pH range in the presence of GFH must have at least in part been attributable to precipitation. A calcium concentration of 15.3 mmol/L in solutions I to N resulted in a molar Ca:DTPMP ratio of about 3200:1. This very high ratio was not investigated in Experiment 3, but given the tendency of the results in Table 5, it seems possible that above a certain molar Ca:DTPMP ratio, precipitation will occur even at low DTPMP concentrations. Furthermore, since orthophosphate was present in all solutions, the precipitation of hydroxyapatite (Ca_5_(PO_4_)_3_OH) was likely at pH > 8 (calculations performed in PHREEQC). Therefore, the enhanced elimination for the solutions I, K, and L at pH > 8 can be ascribed to the precipitation of hydroxyapatite and/or Ca-DTPMP complexes. Both of these precipitates would lead to an elimination of organic phosphorus, either by direct precipitation (Ca-DTPMP complexes) or adsorption on the precipitate (hydroxyapatite) [48]. In the absence of calcium but with magnesium present (solution J), the observed precipitation was shifted towards the more alkaline pH range (pH > 10), which is consistent with the observation that in the presence of GFH at pH > 10 an increased removal occurred. Calculations performed with PHREEQC predict precipitation of Mg(OH)_2_, which is a known adsorbent for phosphorus compounds [49], from pH 10 onwards. 

With additional (hydrogen-)carbonate ions (solutions M and N), a stronger removal and, thus, a stronger precipitation occurred, compared with the previously discussed solutions (without GFH, see Figure 4d and Figure 5d). Calculations performed with PHREEQC, without taking DTPMP into account, showed that the solubility limit of CaCO_3_, dolomite (CaMg(CO_3_)_2_), and hydroxyapatite was exceeded in solutions M and N from pH 7 onwards. Since no precipitation occurred at pH 7, this precipitation must have been impeded by the phosphonate. Above pH 8, however, these three compounds could be precipitated and all of them, as possible adsorbents, could have led to the removal of phosphorus [16,48,50,51,52]. In keeping with the enhanced precipitation observed at pH > 8, elimination also increased in the batches with GFH at higher pH values (Figure 4c and Figure 5c).

The behavior of solution N, the near-complete synthetic replica of the membrane concentrate, and the real membrane concentrate were very similar. Therefore, it can be concluded that all ions that strongly influence phosphonate adsorption were considered in the replica. In conclusion, the competing effect of anions present in the membrane concentrate on phosphonate adsorption can be ranked as follows: HCO_3_^–^ >> SO_4_^2–^, NO_3_^–^. Particularly at pH values > 8, Ca^II^ has a positive effect on phosphorus removal, mainly due to precipitation. The same applies to Mg^II^ at pH values > 10. The influence of the investigated ions on adsorption of DTPMP and PO_4_-P is nearly identical.

## 4. Conclusions

The influence of different compounds in membrane concentrate on the adsorption of phosphonates and phosphate on GFH was investigated. Of all phosphonates tested, HEDP was the phosphonate with the lowest calcium tolerance (precipitation already at a molar Ca:HEDP ratio of 2:1 after seven days contact time). The calcium already contained in the GFH plays an essential role in the elimination process, as it can be re-dissolved, causing a positive effect on the elimination of phosphonates. A further increase in Ca^II^ concentration also caused precipitation of the phosphonates NTMP and DTPMP at pH values > 8, likely as Ca-phosphonate complexes. NTMP and DTPMP showed a similar adsorption behavior, but the solubility of Ca-NTMP complexes was lower than that of Ca-DTPMP complexes. Experiments with membrane concentrate and its synthetic replicas showed that HCO_3_^–^ has a competing effect on phosphorus adsorption, whereas the influence of SO_4_^2–^ and NO_3_^–^ is negligible. Up to pH 8, the presence of Ca^II^ has a positive effect on adsorption, probably due to the formation of ternary complexes. The presence of Ca^II^ (at pH > 8) and Mg^II^ (at pH > 10) led to the formation of precipitates that served as adsorbents for phosphorus compounds, either through direct precipitation of Ca-phosphonate complexes, or through the formation of inorganic precipitates of calcium, magnesium, and phosphate. An additional presence of (hydrogen-)carbonate ions resulted in precipitation of CaCO_3_ and/or dolomite, which also acted as adsorbents for phosphorus compounds. The influence of the investigated ions on the adsorption of DTPMP and PO_4_-P is nearly identical. It can be assumed that membrane concentrate with its high content of Ca^2+^ and Mg^2+^ is well suited for treatment with GFH. Since Ca^II^ is re-dissolved from GFH, future experiments should examine whether sufficient phosphonate is still removed after multiple use. In addition, it is important to investigate whether the precipitates interfere with the adsorption or regeneration process.

## Figures and Tables

**Table 1:** Composition of the solutions. A-N: synthetic replicas, MC: membrane concentrate.

		Solutions														
Compound	Unit	A	B	C	D	E	F	G	H	I	J	K	L	M	N	MC
DTPMP-P	mg/L	0.74	-	0.74	0.74	0.74	0.74	0.74	0.74	0.74	0.74	0.74	0.74	0.74	0.74	0.74
PO_4_-P	mg/L	-	0.53	0.53	0.53	0.53	0.53	0.53	0.53	0.53	0.53	0.53	0.53	0.53	0.53	0.53
NO_3_^-^	mg/L	-	-	-	67.7	-	-	-	67.7	-	-	-	-	-	67.7	67.7
SO_4_^2–^	mg/L	-	-	-	-	474	-	474	474	-	-	-	474	474	474	474
HCO_3_^–^	mg/L	-	-	-	-	-	1620	1620	1620	-	-	-	-	1620	1620	1620
Ca^2+^	mg/L	-	-	-	-	-	-	-	-	613	-	613	613	613	613	613
Mg^2+^	mg/L	-	-	-	-	-	-	-	-	-	75.0	75.0	75.0	75.0	75.0	75.0

**Table 2:** Different concentrations of phosphonates used in Experiment 1.

	Phosphonate		Phosphonate-P
	mg/L	µmol/L	mg/L
HPAA	16.1	103	3.19
PBTC	16.1	59.6	1.85
HEDP	16.1	78.1	4.84
NTMP	16.1	53.8	5.00
EDTMP	16.1	36.9	4.57
DTPMP	16.1	28.1	4.35

**Table 3:** Different concentrations of phosphonates used in Experiment 3.

	Phosphonate		Phosphonate-P
	mg/L	µmol/L	mg/L
NTMP	3.22	10.8	1.0
	16.1	53.8	5.0
	32.2	108	10
	80.5	269	25
DTPMP	3.22	5.61	0.9
	16.1	28.1	4.4
	32.2	56.1	8.7
	80.5	140	22

**Table 4:** Removal of NTMP by precipitation at different calcium concentrations and pH values (T = 20 °C; t_c_ = 7 d; no GFH added). Standard deviations are shown in Table S1.

	NTMP [mg/L]											
	3.22	16.1	32.2	80.5	3.22	16.1	32.2	80.5	3.22	16.1	32.2	80.5
	Calcium [mmol/L]				Removal at pH 8 [%]				Removal at pH 9 [%]			
Ca:NTMP 0:1	0.00	0.00	0.00	0.00	1	0	1	0	3	0	1	2
Ca:NTMP 1:1	0.01	0.05	0.11	0.27	3	0	2	1	−1	0	1	2
Ca:NTMP 2:1	0.02	0.11	0.22	**0.54**	1	0	1	1	0	1	2	**91**
Ca:NTMP 5:1	0.05	0.27	**0.54**	1.35	2	1	2	99	4	2	**78**	99
Ca:NTMP 10:1	0.11	**0.54**	1.08	2.69	1	3	95	99	4	**64**	100	100
Ca:NTMP 25:1	0.27	1.35	2.69	6.73	1	94	99	100	3	97	100	100
Ca:NTMP 60:1	0.65	3.23	6.46	16.1	0	97	99	100	2	99	100	100

**Table 5:** Removal of DTPMP by precipitation at different calcium concentrations and pH values (T = 20 °C; t_c_ = 7 d; no GFH added). Standard deviations are shown in Table S2.

	DTPMP [mg/L]											
	3.22	16.1	32.2	80.5	3.22	16.1	32.2	80.5	3.22	16.1	32.2	80.5
	Calcium [mmol/L]				Removal at pH 8 [%]				Removal at pH 9 [%]			
Ca:DTPMP 0:1	0.00	0.00	0.00	0.00	−2	−1	−1	−1	−2	0	2	−1
Ca:DTPMP 1:1	0.01	0.03	0.06	0.14	−1	1	−4	−1	2	0	4	0
Ca:DTPMP 2:1	0.01	0.06	0.11	0.28	0	−1	−4	−1	−1	0	−2	0
Ca:DTPMP 5:1	0.03	0.14	0.28	**0.70**	0	1	0	0	−1	0	−1	**62**
Ca:DTPMP 10:1	0.06	0.28	0.56	**1.40**	0	0	0	**74**	−4	0	47	91
Ca:DTPMP 25:1	0.14	**0.70**	**1.40**	3.51	−1	−1	**56**	90	−1	**0**	88	94
Ca:DTPMP 60:1	0.34	1.68	3.37	8.42	0	54	84	93	−6	85	92	96

**Table 6:** Change in calcium concentration in the absence of phosphonates (T = 20 °C; pH = 8 and 9; t_c_ = 7 d; no GFH added). Ca_target_: target starting concentration, Ca_start_: actual starting concentration, Ca_end_: final concentration, Deviation: Ca_start_/Ca_end_.

pH_start_	pH_end_	Ca_target_ [mmol/L]	Ca_start_ [mmol/L]	Ca_end_ [mmol/L]	Deviation [%]
8.01	8.04	3.51	3.44	3.42	0.6
7.99	8.00	6.46	6.35	6.40	−0.8
8.01	8.04	6.73	6.80	7.00	−2.9
8.00	8.03	8.42	8.40	8.20	2.4
8.00	8.03	16.10	15.90	16.00	−0.6
9.01	9.01	3.51	3.60	3.64	−1.0
8.98	8.99	6.46	6.30	6.10	3.2
9.01	9.02	6.73	6.50	6.40	1.5
8.97	8.96	8.42	8.45	8.50	−0.6
8.99	8.97	16.10	15.60	15.35	1.6

## Supplementary Materials

The following are available online, Table S1: Removal of NTMP by precipitation at different calcium concentrations and pH values with standard deviations, Table S2: Removal of DTPMP by precipitation at different calcium concentrations and pH values with standard deviations.
