# Supplementary material for: Batch Studies of Phosphonate and Phosphate Adsorption on Granular Ferric Hydroxide (GFH) with Membrane Concentrate and Its Synthetic Replicas
Source: Molecules. 2020 Nov 9;25(21):5202. doi: 10.3390/molecules25215202 (PMC7664883; doi:10.3390/molecules25215202)
Supplement: Supplementary file 1 [file molecules-25-05202-s001.pdf]

molecules

MDPI

Supplementary Material

Batch Studies of Phosphonate and Phosphate Adsorption on Granular Ferric Hydroxide (GFH) with Membrane Concentrate and Its Synthetic Replicas

Tobias Reinhardt, Adriana Noelia Veizaga Campero, Ralf Minke, Harald Schönberger and Eduard Rott

Institute for Sanitary Engineering, Water Quality and Solid Waste Management (ISWA), University of Stuttgart, Bandtäle 2, 70569 Stuttgart, Germany

1. Standard Deviations

Experiment 3 was carried out in a duplicate approach. Tables S1 and S2 show the sample standard deviations of the results. The sample standard deviation was calculated according to Equation S.1, where N is the number of observations on the sample, x_i the observed sample value, and $\bar{x}$ the mean value of these observations.

$$s = \sqrt{\frac{1}{N-1}\sum_{i=1}^{N}(x_i - \bar{x})^2}, \tag{S.1}$$

Table S1: Removal of NTMP by precipitation at different calcium concentrations and pH values with standard deviations (T = 20 °C; t_c = 7 d; no GFH added).

	**NTMP [mg/L]**							
	**3.22**	**16.1**	**32.2**	**80.5**	**3.22**	**16.1**	**32.2**	**80.5**
	**Removal at pH 8 [%]**				**Removal at pH 9 [%]**			
Ca:NTMP 0:1	0.6±1.1	-0.1±0.5	1.1±0.0	0.0±0.4	2.6±0.3	0.2±0.2	0.7±0.7	2.4±1.0
Ca:NTMP 1:1	2.6±1.8	0.3±0.1	1.5±0.1	1.1±0.2	-1.2±0.1	0.2±0.3	0.8±0.2	2.2±0.3
Ca:NTMP 2:1	0.9±0.5	0.2±0.5	1.4±0.1	0.8±0.2	-0.3±0.4	1.4±0.6	1.9±0.2	91.3±0.2
Ca:NTMP 5:1	1.5±0.2	1.2±0.2	1.7±0.1	98.7±0.6	4.3±0.8	1.6±1.3	78.2±1.9	99.4±0.3
Ca:NTMP 10:1	0.7±0.2	2.6±2.2	95.4±2.1	99.5±0.5	3.6±1.1	63.7±0.4	99.6±0.2	99.5±0.1
Ca:NTMP 25:1	1.1±2.4	94.0±0.2	99.0±0.0	99.7±0.1	2.7±0.6	97.0±1.9	100.1±0.2	100.1±0.3
Ca:NTMP 60:1	0.3±0.4	96.6±2.2	99.4±0.0	99.6±0.0	2.0±0.4	98.6±0.7	100.4±0.0	100.1±0.0

Table S2: Removal of DTPMP by precipitation at different calcium concentrations and pH values with standard deviations (T = 20 °C; t_c = 7 d; no GFH added).

	**DTPMP [mg/L]**							
	**3.22**	**16.1**	**32.2**	**80.5**	**3.22**	**16.1**	**32.2**	**80.5**
	**Removal at pH 8 [%]**				**Removal at pH 9 [%]**			
Ca:DTPMP 0:1	-1.5±0.6	-1.1±1.3	-1.2±1.0	-0.6±0.4	-1.6±0.1	0.1±0.1	1.8±2.3	-0.6±0.2
Ca:DTPMP 1:1	-1.4±0.4	1.4±1.5	-4.0±1.1	-1.3±0.7	1.7±4.8	-0.3±0.3	3.7±3.3	-.03±0.1
Ca:DTPMP 2:1	-0.1±0.6	-0.8±1.5	-3.9±0.3	-0.8±0.4	-1.5±0.0	0.2±0.3	-1.5±0.0	-0.3±0.5
Ca:DTPMP 5:1	0.0±1.2	1.0±1.4	0.5±1.5	0.0±0.3	-1.4±0.1	-0.3±0.5	-1.5±1.3	61.9±1.9
Ca:DTPMP 10:1	0.3±0.4	0.0±0.7	-0.1±0.0	73.8±0.3	-4.5±3.0	0.3±0.3	47.0±0.3	91.2±0.3
Ca:DTPMP 25:1	-1.2±0.7	-0.6±3.6	55.6±1.4	90.3±0.1	-1.2±1.6	0.3±0.3	87.7±0.6	94.3±0.1
Ca:DTPMP 60:1	0.0±0.4	54.2±0.2	83.7±0.3	93.3±0.6	-6.2±2.4	84.7±0.2	92.2±0.1	95.6±0.1
